# Acute hypoxia‐reoxygenation and vascular oxygen sensing in the chicken embryo

**DOI:** 10.14814/phy2.13501

**Published:** 2017-11-17

**Authors:** Riazuddin Mohammed, Carlos E. Salinas, Dino A. Giussani, Carlos E. Blanco, Angel L. Cogolludo, Eduardo Villamor

**Affiliations:** ^1^ Department of Pediatrics Maastricht University Medical Center (MUMC+) School for Oncology and Developmental Biology (GROW) Maastricht the Netherlands; ^2^ Instituto Boliviano de Biología de Altura Facultad de Medicina Universidad Mayor de San Andrés La Paz Bolivia; ^3^ Department of Physiology Development & Neuroscience University of Cambridge Cambridge United Kingdom; ^4^ Department of Neonatology National Maternity Hospital Dublin Ireland; ^5^ Department of Pharmacology School of Medicine Universidad Complutense de Madrid Centro de Investigaciones Biomédicas en Red de Enfermedades Respiratorias (CIBERES) Instituto de Investigación Sanitaria Gregorio Marañón (IiSGM) Madrid Spain

**Keywords:** Oxygen signaling, hypoxia, hyperoxia, endothelium, chicken embryo

## Abstract

Fetal/perinatal hypoxia is one of the most common causes of perinatal morbidity and mortality and is frequently accompannied by vascular dysfunction. However, the mechanisms involved have not been fully delineated. We hypothesized that exposure to acute hypoxia‐reoxygenation induces alterations in vascular O_2_ sensing/signaling as well as in endothelial function in the chicken embryo pulmonary artery (PA), mesenteric artery (MA), femoral artery (FA), and ductus arteriosus (DA). Noninternally pipped 19‐day embryos were exposed to 10% O_2_ for 30 min followed by reoxygenation with 21% O_2_ or 80% O_2_. Another group was constantly maintained at 21% O_2_ or at 21% O_2_ for 30 min and then exposed to 80% O_2_. Following treatment, responses of isolated blood vessels to hypoxia as well as endothelium‐dependent (acetylcholine) and ‐independent (sodium nitroprusside and forskolin) relaxation were investigated in a wire myograph. Hypoxia increased venous blood lactate from 2.03 ± 0.18 to 15.98 ± 0.73 mmol/L (*P* < 0.001) and reduced hatchability to 0%. However, ex vivo hypoxic contraction of PA and MA, hypoxic relaxation of FA, and normoxic contraction of DA were not significantly different in any of the experimental groups. Relaxations induced by acetylcholine, sodium nitroprusside, and forskolin in PA, MA, FA, and DA rings were also similar in the four groups. In conclusion, exposure to acute hypoxia‐reoxygenation did not affect vascular oxygen sensing or reactivity in the chicken embryo. This suggests that direct effects of acute hypoxia‐reoxygenation on vascular function does not play a role in the pathophysiology of hypoxic cardiovascular injury in the perinatal period.

## Introduction

Prenatal development takes place in an environment, that is, relatively hypoxic compared with the postnatal environment (Maltepe and Saugstad 2009). Besides this physiological hypoxia, the fetus can be exposed to acute, intermittent, or chronic hypoxic challenges, which if severe can lead to injury or death (Maltepe and Saugstad 2009; Vento et al. 2012; Giussani 2016). Reoxygenation following exposure to hypoxia may exacerbate the hypoxic damage (Maltepe and Saugstad 2009; Vento et al. 2012). Production of reactive O_2_ species (ROS) is a key component of the pathogenesis of hypoxia‐reoxygenation injury (Li and Jackson 2002; Maltepe and Saugstad 2009). Vascular endothelial cells are the primary target for ROS generated by themselves immediately after reoxygenation/reperfusion and, at later times, by adherent leukocytes (Sheridan et al. 1991; Li and Jackson 2002; Ng et al. 2002). In addition, ROS‐mediated injury of vascular smooth muscle cells may amplify the damage (Mohazzab‐H et al. 1996; Ng et al. 2002).

The chicken embryo is a valuable model for the study of the pathophysiological effects of acute and chronic prenatal hypoxia (Villamor et al. 2004; Giussani et al. 2007; Agren et al. 2008; Zoer et al. 2009; Lindgren et al. 2010, 2011; Salinas et al. 2010, 2011, 2014; Moonen et al. 2012; Itani et al. 2016). Hypoxia is easily induced by incubating the embryonated egg in a low O_2_ environment and its direct effects on the fetus can be isolated without additional effects on the maternal or placental physiology, as occurs in mammals. Either in the mammalian fetus or the chicken embryo the cardiovascular defense responses to acute hypoxia are conserved and include a redistribution of the cardiac output away from the peripheral circulation toward higher priority organs such as the heart, brain, and adrenal glands (Giussani et al. 1993; Mulder et al. 2002; Giussani 2016). An important component of hypoxia‐induced blood flow redistribution is triggered by a carotid chemoreflex and maintained by the release of vasoactive agents into the fetal circulation, such as catecholamines, vasopressin, and neuropeptide Y (Giussani et al. 1993; Mulder et al. 2002; Fletcher et al. 2006; Giussani 2016). In addition, recent discoveries suggest that chemoreflex and endocrine vasoactive mechanisms are complemented by an increased local vascular oxidant tone (Thakor et al. 2010). The latter can be promoted by the interaction between nitric oxide (NO) and ROS generated during acute hypoxia, such as the superoxide anion (O_2_.^−^) (Kane et al. 2012, 2014; Thakor et al. 2015). Finally, hypoxia‐mediated redistribution of blood flow can also be influenced by a direct vascular effect of hypoxia, which may promote contraction or relaxation depending on the vascular bed (Agren et al. 2007; Greyner and Dzialowski 2008; Cogolludo et al. 2009; Zoer et al. 2009, 2010; van der Sterren et al. 2011; Moreno et al. 2014; Van der Sterren et al. 2014; Brinks et al. 2016).

Hypoxia‐induced contraction of the isolated pulmonary artery (PA)(Zoer et al. 2009; van der Sterren et al. 2011; Moreno et al. 2014) and the mesenteric artery (MA) (Brinks et al. 2016), as well as hypoxia‐induced relaxation of the isolated femoral artery (FA) (Zoer et al. 2010; van der Sterren et al. 2011) and the ductus arteriosus (DA) (Agren et al. 2007; Greyner and Dzialowski 2008; Cogolludo et al. 2009; van der Sterren et al. 2011; Van der Sterren et al. 2014) have been described in chicken embryos**.** However, whether these direct effects of hypoxia on vasomotor tone is modified by acute preexposure to hypoxia is completely unknown. In this study, we hypothesized that exposure to acute hypoxia‐reoxygenation induces alterations in O_2_ sensing/signaling as well as in endothelial function in chick embryo blood vessels. To test our hypothesis, we exposed 19‐day chicken embryos to 30 min of hypoxia (10% O_2_) followed by 15 min of reoxygenation with 21 or 80% O_2_. Immediately after the hypoxia‐reoxygenation challenge, rings from the PA, MA, FA, and DA were mounted in a wire myograph in order to test ex vivo response to hypoxia‐reoxygenation and endothelium‐dependent and ‐independent relaxation.

## Methods

### Incubation of chicken embryos and exposure to hypoxia‐reoxygenation

All experimental procedures were carried out in accordance with the Dutch Law on Animal Experimentation and the European Directive for the Protection of Vertebrate Animals Used for Experimental and Other Scientific Purposes (86/609/EU) and approved by the Committee on Animal Experimentation of the University of Maastricht. Fertilized eggs from White Leghorn chickens were incubated at 37.8°C, 45% humidity, and atmospheric O_2_ concentration and rotated once per hour over an angle of 90° (Incubator model 25HS, Masalles Comercial, Spain). On day 19 of the 21 days of incubation, the absence of internal pipping was assessed by transillumination and the eggs were transferred to a plexiglas chamber in which the same conditions of humidity and temperature were maintained but O_2_ concentration was varied between 10 and 80%. O_2_ level in the chamber was continuously monitored with a DrDAQ O_2_ sensor (Pico Technology, United Kingdom). Eggs were exposed to 10% O_2_ for 30 min followed by 15 of reoxygenation with 21% O_2_ (HypOx‐NormOx group) or 80% O_2_ (HypOx‐HyperOx). A group of eggs was maintained at 21% O_2_ for 45 min (NormOx‐NormOx) and a group of eggs was maintained at 21% O_2_ for 30 min and then exposed to 80% O_2_ for 15 min (NormOx‐HyperOx). Following the experimental exposure, the embryos were extracted from the egg and a blood sample was drawn from the jugular vein and immediately used for blood lactate determination with a portable analyzer (Lactate Scout, SensLab GmbH, Leipzig, Germany). Then the embryos were killed by decapitation.

In a separate group of experiments, 12 eggs from each experimental group (NormOx‐NormOx, NormOx‐HyperOx, HypOx‐NormOx, and HypOx‐HyperOx) were transferred to hatching baskets and incubated at 37.8°C, 45% humidity, and atmospheric O_2_ concentration until hatch. On the 22nd day of incubation all eggs which did not hatch were opened and examined.

### Dissection of vessels and measurement of arterial reactivity

Embryos were placed on the dorsal side on a Petri‐dish coated with silicon and a midline laparotomy and sternotomy were performed. With the aid of a dissecting microscope, the DA, the caudomedial intrapulmonary artery, the cranial MA and the FA were carefully dissected as previously described (Agren et al. 2007; Cogolludo et al. 2009; Zoer et al. 2009, 2010; van der Sterren et al. 2011; Moreno et al. 2014; Van der Sterren et al. 2014; Brinks et al. 2016). Two stainless steel wires (diameter 40 *μ*m) were inserted into the lumen of the vessels, which were mounted as a 1.7–2 mm length ring segment between an isometric force transducer and a displacement device in a myograph (Danish Myo Technology A/S model 610M, Aarhus, Denmark). The myograph organ bath (5 mL vol) was filled with Krebs‐Ringer bicarbonate buffer (KRB, composition in mmol/L: NaCl, 118.5; KCl, 4.75; MgSO_4_ •7H_2_O, 1.2; KH_2_PO_4_, 1.2; NaHCO_3_, 25.0; CaCl_2_, 2.5; glucose, 5.5) maintained at 39°C and aerated with 21% O_2_‐74% N_2_‐5% CO_2_ (Po_2_ ~ 19.2 kPa, measured with an ABL 510 blood gas analyzer, Radiometer Copenhagen, Denmark). After an equilibration period of 30 min, the vessels were distended to their individual optimal diameter, which evoked a resting tension corresponding to a transmural pressure of 20 mmHg. This pressure corresponds to the mean arterial blood pressure reported in 19‐day chicken embryos and elicited the highest contractile response to KCl, as determined in previous experiments (Agren et al. 2007; Cogolludo et al. 2009; Zoer et al. 2009, 2010; van der Sterren et al. 2011; Moreno et al. 2014; Van der Sterren et al. 2014; Brinks et al. 2016). After 30 min of incubation under resting tension, a control reference contraction was elicited by raising the K^+^ concentration of the buffer (62.5 mmol/L) in exchange for Na^+^.

### Response to acute hypoxia

**Figure 1:**
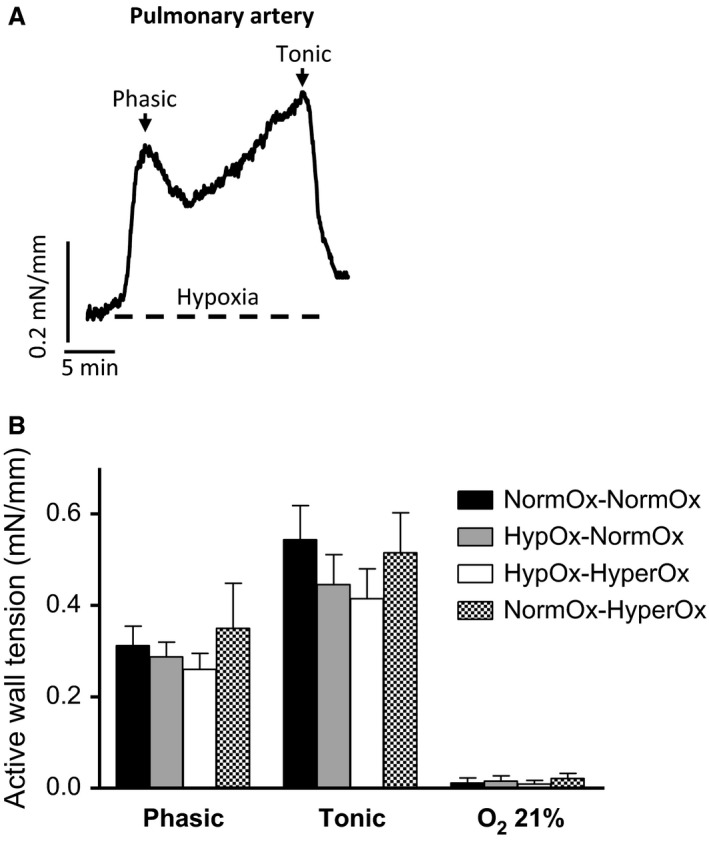
(A) Representative original tracing showing the response of an isolated 19‐d chicken embryo caudomedial intrapulmonary artery to 15 min of hypoxia followed by reoxygenation. Hypoxia was induced by switching the gas mixture aerating the organ bath from 21% O_2_‐74% N_2_‐5% CO_2_ (Po_2_~19.2 kPa) to 95% N_2_‐5% CO_2_ (Po_2_~2.5 kPa). (B) Response to hypoxia of pulmonary artery rings from 19‐day chicken embryos exposed to in ovo acute hypoxia‐reoxygenation. Eggs were exposed to 10% O_2_ for 30 min followed by 15 min of reoxygenation with 21% O_2_ (HypOx‐NormOx group) or 80% O_2_ (HypOx‐HyperOx). A group of eggs was maintained at 21% O_2_ for 45 min (NormOx‐NormOx) and a group of eggs was maintained at 21% 0_2_ for 30 min and then exposed to 80% O_2_ for 15 min (NormOx‐HyperOx group).Results are shown as mean ± SE of measurements in 6–9 embryos.

The effects of acute hypoxia on MA, PA, and DA wall tension were studied in quiescent (i.e., without pretone), endothelium‐intact rings. In FA rings, pretone was induced by norepinephrine (NE, 10 μmol/L). To induce hypoxia, the organ chambers were wrapped in cling film and the gas mixture aerating the organ bath was switched from 21% O_2_‐74% N_2_‐5% CO_2_ (Po_2_ ~ 19.2 kPa) to 95% N_2_‐5% CO_2_ (Po_2_ ~2.5 kPa). MA, PA, and FA rings were exposed to a normoxia‐15 min hypoxia‐normoxia challenge, whereas DA rings were exposed to a hypoxia‐15 min normoxia‐hypoxia challenge (Fig. 1).

### Endothelium‐dependent and ‐independent relaxation

In order to analyze vascular relaxation MA, FA, and DA rings were contracted with NE (10 *μ*mol/L) and PA rings were contracted with KCl (62.5 mmol/L). Previous experiments showed that these stimuli elicited a stable contraction of the vessels. Once reached a stable tone, concentration–response curves to the muscarinic receptor agonist acetylcholine (ACh, 10 nmol/L–0.1 mmol/L), the nitric oxide (NO) donor sodium nitroprusside (SNP, 10 nmol/L–0.1 mmol/L), and the adenylate cyclase activator forskolin (10 nmol/L–10 *μ*mol/L) were performed. After determining the response to each mediator, the vessels were rinsed three times with fresh KRB buffer and allowed to recover to baseline.

### Drugs and solutions

Solutions containing different concentrations of K^+^ were prepared by replacing part of the NaCl of the KRB buffer by an equimolar amount of KCl. All the drugs were obtained from Sigma (St. Louis, MO) and were dissolved initially in distilled deionized water (except forskolin in ethanol) to prepare adequate stock solutions and further dilutions were also made in deionized water. The final bath concentration of ethanol did not exceed 0.1% and had no effect on activity.

### Data analysis

Data are shown as mean ± SE of measurements in *n* embryos. Contractions are expressed in terms of active wall tension (mN/mmol/L, calculated as the force divided by twice the length of the segment) or as a percentage of the reference contraction to KCl (62.5 mmol/L) performed for each individual ring at the beginning of the experiment. The relaxant responses are expressed as the percentage of reduction in the contraction induced by NE or KCl. Sensitivity (expressed as pEC_50_^ ^= ‐log EC_50_) and maximal relaxation (*E*_max_) to agonists was determined by fitting individual concentration–response data to a nonlinear sigmoidal regression curve. Differences between mean values were assessed by one‐way ANOVA followed by Bonferroni's post hoc *t*‐test. Differences were considered significant at a *P* < 0.05. All analyses were performed using GraphPad Prism (version 5.00 for Windows, GraphPad Software, San Diego California, www.graphpad.com).

## Results

The 19‐day noninternally pipped chicken embryos incubated under 21% O_2_ (NormOx‐NormOx) had a venous blood lactate concentration of 2.03 ± 0.18 mmol/L (*n* = 8). In contrast, blood lactate was 15.98 ± 0.73 mmol/L in the embryos of the HypOx‐NormOx group (*n* = 8; *P* < 0.001 vs. NormOx‐NormOx) and 14.60 ± 0.50 mmol/L in the embryos of the HypOx‐HyperOx group (*n* = 8; *P* < 0.001 vs. NormOx‐NormOx). The embryos of the NormOx‐HyperOx group showed a blood lactate concentration (2.12 ± 0.19 mmol/L, *n* = 8) that was not significantly different when compared with the NormOx‐NormOx group. During the manipulation that accompanied venous puncture, the embryos from the NormOx‐NormOx group and the NormOx‐HyperOx group showed reactive head and wing movements. These movements were markedly reduced in number and amplitude in the embryos from the HypOx‐NormOx and the HypOx‐HyperOx groups. Heart beating was observed during dissection in the four experimental groups.

When unopened eggs of the NormOx‐NormOx group (*n* = 12) and the NormOx‐HyperOx group (*n* = 12) were returned to the incubator and maintained under normoxic conditions, hatchability at day 21 was 100%. In contrast, none of the embryos of the HypOx‐NormOx group (*n* = 12) and the HypOx‐HyperOx group (*n* = 12) hatched. At day 22, unhatched eggs were opened and the presence of a dead embryo was verified in all cases.

### Vascular reactivity

**Figure 2:**
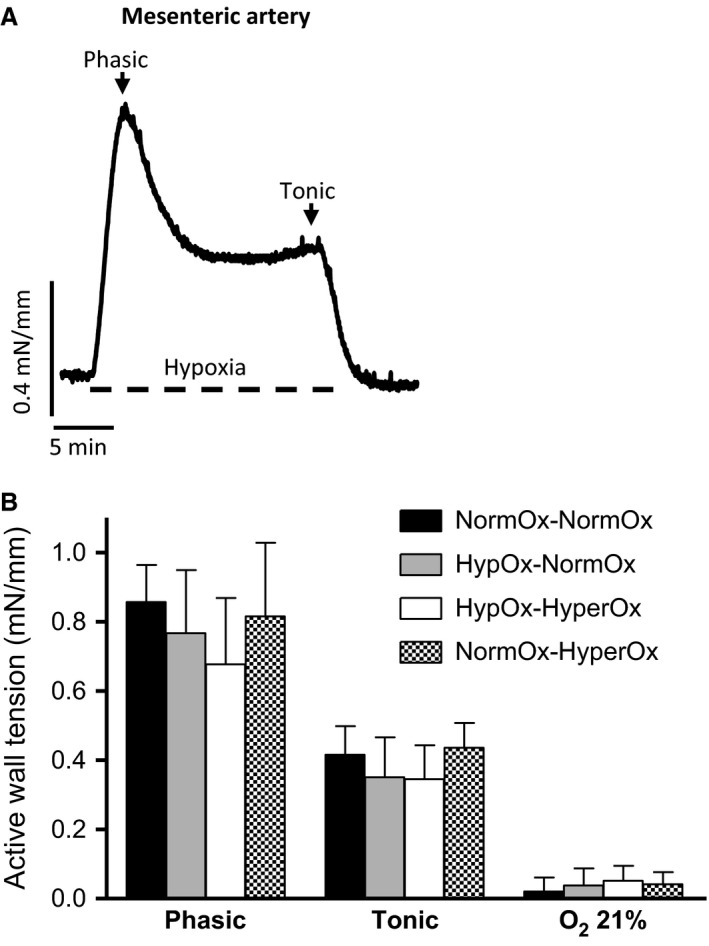
(A) Representative original tracing showing the response of an isolated 19‐d chicken embryo cranial mesenteric artery to hypoxia for 15 min. Hypoxia was induced by switching the gas mixture aerating the organ bath from 21% O_2_‐74% N_2_‐5% CO_2_ (Po_2_~19.2 kPa) to 95% N_2_‐5% CO_2_ (Po_2_~2.5 kPa). (B) Response to hypoxia of mesenteric artery rings from 19‐day chicken embryos exposed in ovo to acute hypoxia‐reoxygenation. Eggs were exposed to 10% O_2_ for 30 min followed by 15 min of reoxygenation with 21% O_2_ (HypOx‐NormOx group) or 80% O_2_ (HypOx‐HyperOx). A group of eggs was maintained at 21% O_2_ for 45 min (NormOx‐NormOx) and a group of eggs was maintained at 21% 0_2_ for 30 min and then exposed to 80% O_2_ for 15 min (NormOx‐HyperOx). Results are shown as mean ± SE of measurements in 6–9 embryos.

When PA (Fig. 1A) and MA (Fig. 2A) rings were exposed to 15 min of hypoxia (in the absence of pretone), they developed a rapid contraction (from here on referred to as phasic contraction) which peaked within 2–4 min. Tension then fell, reaching a nadir within 8–10 min, after which it tended to increase gradually. This second phase is from now on named tonic contraction. The amplitude of the phasic contraction was measured as the initial peak in tension development occurring within the first 4 min, whereas the amplitude of the tonic contraction was taken to be the level of tension above baseline measured after 20 min of hypoxia, immediately before reoxygenation.

**Figure 3:**
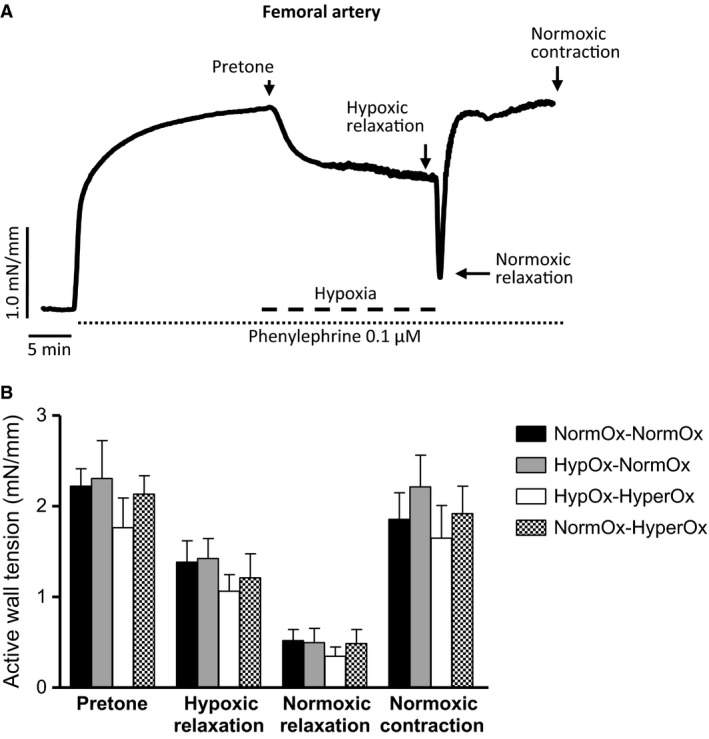
(A) Representative original tracing showing the response of an isolated 19‐day chicken embryo femoral artery to hypoxia for 15 min. The vessel was precontracted with norepinephrine (1 *μ*mol/L) and hypoxia was induced by switching the gas mixture aerating the organ bath from 21% O_2_‐74% N_2_‐5% CO_2_ (Po_2_ ~ 19.2 kPa) to 95% N_2_‐5% CO_2_ (Po_2_~2.5 kPa). (B) Response to hypoxia of femoral artery rings from 19‐day chicken embryos exposed in ovo to acute hypoxia‐reoxygenation. Eggs were exposed to 10% O_2_ for 30 min followed by 15 min of reoxygenation with 21% O_2_ (HypOx‐NormOx) or 80% O_2_ (HypOx‐HyperOx). A group of eggs was maintained at 21% O_2_ for 45 min (NormOx‐NormOx) and a group of eggs was maintained at 21% 0_2_ for 30 min and then exposed to 80% O_2_ for 15 min (NormOx‐HyperOx). Results are shown as mean ± SE of measurements in 6–9 embryos.

In FA rings contracted with NE (1 *μ*mol/L), hypoxia induced a sustained relaxation (Fig. 3A). This relaxation was preceded by a transient contraction in ~50% of the vessels. Reoxygenation with 21% O_2_ caused a transient additional relaxation before the recovery of normoxic levels of tension (Fig. 3A).

**Figure 4:**
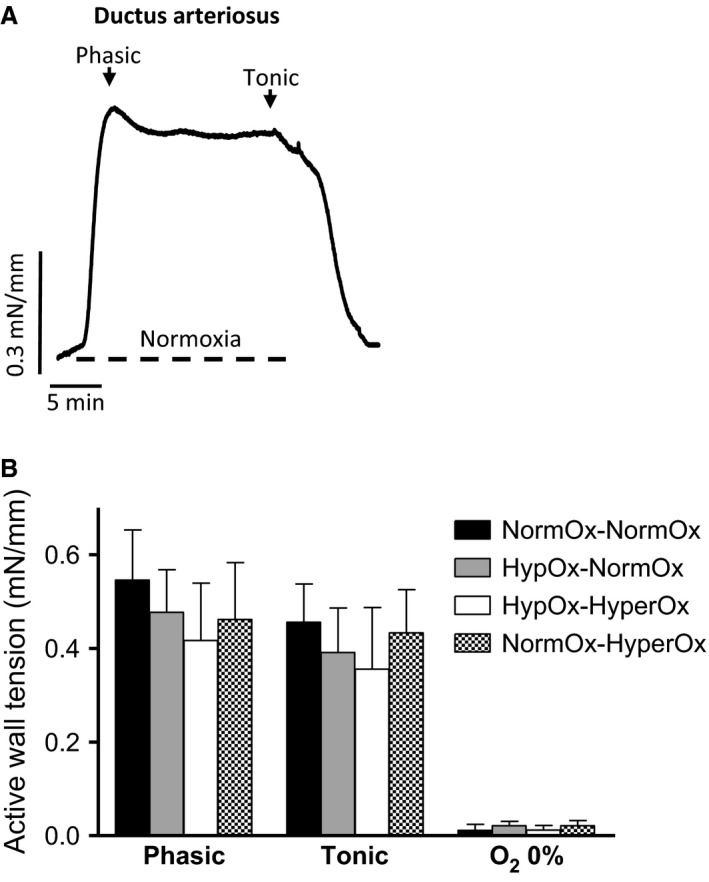
(A) Representative original tracing showing the response of an isolated 19‐d chicken embryo ductus arteriosus (DA) ring to 15 min of normoxia followed by hypoxia. Normoxia was induced by switching the gas mixture aerating the organ bath from 95% N_2_‐5% CO_2_ (Po_2_~2.5 kPa) to 21% O_2_‐74% N_2_‐5% CO_2_ (Po_2_~19.2 kPa). (B) Response to normoxia of DA rings from 19‐day chicken embryos exposed in ovo to acute hypoxia‐reoxygenation. Eggs were exposed to 10% O_2_ for 30 min followed by 15 min of reoxygenation with 21% O_2_ (HypOx‐NormOx) or 80% O_2_ (HypOx‐HyperOx). A group of eggs was maintained at 21% O_2_ for 45 min (NormOx‐NormOx) and a group of eggs was maintained at 21% 0_2_ for 30 min and then exposed to 80% O_2_ for 15 min (NormOx‐HyperOx). Results are shown as mean ± SE of measurements in 6–9 embryos.

When DA rings incubated under hypoxia were exposed to normoxia, they developed a rapid phasic contraction which peaked within 2–4 min. Tension then fell slightly but was maintained by the end of normoxic exposure (tonic contraction) (Fig. 4A). The amplitude of the phasic contraction was measured as the initial peak in tension development occurring within the first 4 min, whereas the amplitude of the tonic contraction was taken to be the level of tension above baseline measured after 15 min of normoxia.

In ovo exposure to hypoxia followed by reoxygenation with 21 or 80% O_2_ did not affect the response to a cycle of normoxia‐hypoxia‐normoxia in PAs (Fig. 1B), MAs (Fig. 2B), or FAs (Fig. 3B) nor the response to a cycle of hypoxia‐normoxia‐hypoxia in the chicken embryo DA (Fig. 4B). In addition, as shown in Table 1, in ovo hypoxia‐reoxygenation did not affect the response of the vessels to KCl, NE, ACh, SNP, or forskolin.

**Table 1:** Contraction and relaxation of blood vessels from 19‐day chicken embryos exposed to hypoxia‐reoxygenation

Blood vessel	Experimental group	Contraction				Relaxation								
		KCl (62.5 mmol/L)		NE (10 *μ*mol/L)		ACh (10 nmol/L‐0.1 mmol/L)			SNP (10 nmol/L–0.1 mmol/L)			Forskolin (10 nmol/L–10 *μ*mol/L)		
		mN/mmol/L	*n*	mN/mmol/L	*n*	*E*_max_	pEC_50_	*n*	*E*_max_	pEC_50_	*n*	*E*_max_	pEC_50_	*n*
Pulmonary artery	NormOx‐NormOx	0.18 ± 0.03	18	–	–	74.9 ± 5.9	7.07 ± 0.1	6	97.2 ± 6.1	6.31 ± 0.1	6	102.2 ± 8.3	5.82 ± 0.2	6
	HypOx‐NormOx	0.15 ± 0.03	18	–	–	71.3 ± 4.9	6.88 ± 0.2	6	94.3 ± 7.9	6.46 ± 0.2	6	104.3 ± 9.1	5.64 ± 0.2	6
	HypOx‐HyperOx	0.17 ± 0.03	17	‐	‐	77.9 ± 6.2	6.96 ± 0.4	6	96.4 ± 6.3	6.36 ± 0.3	6	97.4 ± 8.3	5.71 ± 0.3	6
	NormOx‐HyperOx	0.14 ± 0.04	17	–	–	70.2 ± 6.4	6.81 ± 0.2	6	101.2 ± 8.1	6.51 ± 0.2	6	96.2 ± 8.7	5.63 ± 0.2	6
Mesenteric artery	NormOx‐NormOx	0.94 ± 0.18	18	0.88 ± 0.11	18	103.2 ± 8.1	6.06 ± 0.2	7	101.4 ± 8.7	6.32 ± 0.1	7	97.2 ± 8.7	6.86 ± 0.3	9
	HypOx‐NormOx	0.78 ± 0.17	19	0.83 ± 0.14	19	96.3 ± 8.2	5.98 ± 0.2	7	96.3 ± 8.8	6.24 ± 0.1	7	106.3 ± 9.5	7.01 ± 0.3	8
	HypOx‐HyperOx	0.89 ± 0.11	17	0.86 ± 0.14	17	106.4 ± 8.3	6.14 ± 0.3	7	107.0 ± 8.6	6.15 ± 0.2	6	103.4 ± 8.8	6.94 ± 0.3	7
	NormOx‐HyperOx	0.88 ± 0.14	17	0.76 ± 0.18	17	102.2 ± 9.1	5.89 ± 0.3	8	106.0 ± 10.2	6.25 ± 0.3	7	95.2 ± 7.8	6.87 ± 0.3	7
Femoral artery	NormOx‐NormOx	1.63 ± 0.18	19	2.21 ± 0.19	19	98.2 ± 10.4	6.96 ± 0.3	6	101.3 ± 8.6	5.91 ± 0.2	8	98.6 ± 9.7	5.96 ± 0.3	6
	HypOx‐NormOx	1.71 ± 0.21	19	1.97 ± 0.22	19	107.3 ± 9.2	6.98 ± 0.3	7	104.2 ± 9.1	5.81 ± 0.3	7	96.3 ± 9.1	5.94 ± 0.2	7
	HypOx‐HyperOx	1.83 ± 0.13	17	2.31 ± 0.26	17	101.9 ± 10.3	6.88 ± 0.3	7	98.2 ± 10.1	5.78 ± 0.2	8	101.4 ± 7.8	6.01 ± 0.3	8
	NormOx‐HyperOx	1.69 ± 0.16	17	2.11 ± 0.20	17	96.2 ± 8.1	7.01 ± 0.3	6	97.3 ± 9.2	5.93 ± 0.3	7	105.2 ± 10.8	6.03 ± 0.3	7
Ductus arteriosus	NormOx‐NormOx	0.38 ± 0.05	19	0.45 ± 0.08	19	93.2 ± 7.4	6.81 ± 0.3	7	98.3 ± 6.4	6.52 ± 0.3	9	97.2 ± 8.7	6.32 ± 0.2	8
	HypOx‐NormOx	0.48 ± 0.06	19	0.44 ± 0.06	19	96.4 ± 10.1	6.91 ± 0.3	7	104.3 ± 8.9	6.54 ± 0.2	8	106.3 ± 9.5	6.24 ± 0.3	7
	HypOx‐HyperOx	0.51 ± 0.06	17	0.52 ± 0.06	17	90.4 ± 7.3	6.72 ± 0.3	6	96.7 ± 8.6	6.45 ± 0.3	7	103.4 ± 8.8	6.33 ± 0.1	8
	NormOx‐HyperOx	0.44 ± 0.05	17	0.47 ± 0.05	17	92.2 ± 8.7	6.78 ± 0.3	6	102.2 ± 9.1	6.37 ± 0.4	8	95.2 ± 7.8	6.39 ± 0.1	7

Eggs were exposed to 10% O_2_ for 30 min followed by 15 min of reoxygenation with 21% O_2_ (HypOx‐NormOx) or 80% O_2_ (HypOx‐HyperOx). A group of eggs was maintained at 21% O_2_ for 45 min (NormOx‐NormOx) and a group of eggs was maintained at 21% 0_2_ for 30 min and then exposed to 80% O_2_ for 15 min (NormOx‐HyperOx). Results are shown as mean ± SE of measurements in *n* embryos. ACh, acetylcholine; NE, norepinephrine; SNP, sodium nitroprusside.

## Discussion

Depending on duration and intensity, periodic hypoxic episodes can exert either detrimental or protective effects (Manukhina et al. 2006). In this study, we characterized a model of acute hypoxia‐reoxygenation in the chicken embryo. In the 19‐day chicken embryo, exposure to 10% O_2_ for 30 min induced a 7.5‐fold increase in venous blood lactate and reduced further hatchability to 0%, indicating the presence of an acute, lethal insult. Nevertheless, this hypoxic insult, followed by reoxygenation with normoxia or hyperoxia, did not significantly affect vascular reactivity since neither ex vivo vascular O_2_ signaling nor relaxation were affected by in ovo hypoxia‐reoxygenation.

Blood vessels sense and respond to changes in local O_2_ tension ranging from moderate changes to severe hypoxia (Weir et al. 2005). The fetal circulation is characterized by high pulmonary vascular resistance (PVR), low systemic vascular resistance, the presence of an additional low resistance vascular system (i.e., the placental vascular bed), and right‐to‐left shunting via the foramen ovale and DA (Weir et al. 2005; Gao and Raj 2010; Dzialowski et al. 2011). Vascular O_2_ sensing plays a key role in maintaining this pattern of circulation as well as in the transition to the postnatal circulation. Thus, the fetal relative hypoxic environment induces pulmonary vasoconstriction and DA relaxation favoring the presence of high PVR and right‐to‐left ductal shunt. At birth, the increase in O_2_ causes relaxation of pulmonary arteries and contraction of the DA, thus diverting blood flow to the lungs (Weir et al. 2005; Gao and Raj 2010; Lakshminrusimha and Saugstad 2016). In addition, episodes of fetal or perinatal acute hypoxia trigger chemoreflex, endocrine, and local redox mechanisms mediating the redistribution of cardiac output (Zoer et al. 2010; Brinks et al. 2016; Rainaldi and Perlman 2016). To the best of our knowledge, our study provides the first experimental evidence on the effects of a previous hypoxic insult on the O_2_ sensitivity of fetal vessels. The four vessels investigated show an exquisite O_2_ sensitivity and share some components of the O_2_ signaling pathway such as the presence of a mitochondrial sensor or the utilization of calcium‐dependent and calcium‐sensitizing pathways (Greyner and Dzialowski 2008; Cogolludo et al. 2009; Zoer et al. 2010; Moreno et al. 2014; Brinks et al. 2016). Nevertheless, and in disagreement with our hypothesis, these mechanisms of vascular O_2_ sensing‐signaling were not affected by preexposure to acute hypoxia‐reoxygenation.

Acute hypoxia results in rapid and significant increases in PA pressure as a result of pulmonary vasoconstriction (Weir et al. 2005; Gao and Raj 2010; Sylvester et al. 2012; Lakshminrusimha and Saugstad 2016). These acute changes often lead to a significant hemodynamic compromise which can persist, despite a return to normoxic conditions (Lakshminrusimha and Saugstad 2016). Moreover, an early hypoxic episode may induce constriction of pulmonary arterioles which in turn causes further right‐to‐left shunting and an increase in the oxygenation defect. Consequently, chronic fetal hypoxia or perinatal hypoxia is frequently associated with persistent pulmonary hypertension of the newborn (Lapointe and Barrington 2011; Lakshminrusimha and Saugstad 2016). In addition, in experimental models and human subjects perinatal exposure to acute hypoxia increased hypoxic pulmonary vasoconstriction as well as the severity of pulmonary hypertension following reexposure to hypoxia in later life (Hampl and Herget 1990; Tang et al. 2000; Papamatheakis et al. 2013). Thus, it has been suggested that perinatal hypoxia can program the lung for exaggerated hypoxic pulmonary vasoconstriction (Papamatheakis et al. 2013; Julian et al. 2015). In contrast, our data suggest that hypoxic pulmonary vasoconstriction is not exacerbated by exposure to transient hypoxia‐reoxygenation. Therefore, it can be speculated that hypoxia‐induced impairment of pulmonary vascular O_2_ signaling is not involved in the pathogenesis of neonatal hypoxemic respiratory failure and pulmonary hypertension. However, it should be taken into account that our experiments were performed in conductance PAs. Although, insights into hypoxic pulmonary vasoconstriction have largely emerged from studies employing rings from conductance PAs, the O_2_ sensing and signal transduction machinery appears to be located in smooth muscle cells of the small precapillary vessels (Sylvester et al. 2012). Whether O_2_ sensing in these cells is affected by exposure to acute perinatal hypoxia warrants further investigation.

Despite the key role of O_2_ in the contraction of the DA, perinatal hypoxia does not appear to delay ductal closure and patency of the DA is not considered as one of the cardiocirculatory consequences of perinatal asphyxia (Lapointe and Barrington 2011; Sehgal et al. 2012; Rainaldi and Perlman 2016). Accordingly, our data show that O_2_ sensing and reactivity of chicken DA remained unaltered after an acute hypoxic insult. Previous studies showed that in ovo chronic hypoxia did not affect O_2_ responsiveness of the DA from chicken embryos older than 19 days (Copeland and Dzialowski 2009; Van der Sterren et al. 2009). Interestingly, we also observed that in ovo exposure to acute hyperoxia (preceded or not by hypoxia) did not either affect normoxic contraction of the DA. In a previous study, we analyzed the effects of a more prolonged in ovo exposure to hyperoxia on DA reactivity. Incubation between day 15 and 19 under 60% O_2_ did not affect normoxic contraction of the 19‐d chicken embryo DA (Van der Sterren et al. 2014). In contrast, Greyner and Dzialowski reported that ex vivo exposure of 19‐d chicken embryo DA to 25% O_2_, sensitized the vessel to O_2_ (Greyner and Dzialowski 2015). Interestingly, in another study of the same group it was observed that exposure to 30% O_2_ from the first day of incubation did not affect normoxic contraction of the DA of noninternally pipped chicken embryos (Copeland and Dzialowski 2009). On the contrary, the DA from the hyperoxia‐incubated 20‐d, internally pipped embryos showed a higher O_2_‐induced contraction than the normoxia‐incubated age‐matched controls (Copeland and Dzialowski 2009). However, it should be taken into account that during internal pipping chicken embryos are using both the lungs and the chorioallantoic membrane as respiratory organs. This establishment of lung respiration appears to be critical for chicken DA closure (Belanger et al. 2008; Copeland and Dzialowski 2009; Dzialowski et al. 2011) and high O_2_ tension in embryos relying on chorioallantoic respiration does not appear to affect the developmental trajectory of DA O_2_ responsiveness or to stimulate DA closure (Copeland and Dzialowski 2009; Van der Sterren et al. 2014).

It is generally accepted that while pulmonary arteries respond to acute hypoxia with vasoconstriction, systemic arteries dilate (Sylvester et al. 2012; Morales‐Cano et al. 2015). Systemic hypoxic vasodilation is a conserved physiological response to hypoxia that matches local blood flow and O_2_ delivery to tissue metabolic demand (Maher et al. 2008). Interestingly, the two systemic arteries investigated in this study showed a diametrically opposite pattern of response to acute hypoxia. Thus, the femoral artery did not respond to hypoxia in the absence of pretone and relaxed when pretone was induced by NE (Zoer et al. 2010; van der Sterren et al. 2011). On the contrary, the mesenteric artery contracted to hypoxia either in the absence or presence of pretone (Brinks et al. 2016). When focusing on the signaling pathways, the presence of mitochondrial electron transport chain inhibitors evoked a marked impairment of hypoxic relaxation in femoral arteries (Zoer et al. 2010), whereas the inhibition only had a modest effect on hypoxic mesenteric contraction (Brinks et al. 2016). Independently of these differences, hypoxia‐reoxygenation did not affect the response of FA and MA rings to acute hypoxia. Accordingly, in a previous study we demonstrated that neither fetal nor perinatal hypoxia affected hypoxic relaxation of carotid arteries from newborn rats (Strackx et al. 2010).

Endothelial damage is a key component in the pathophysiology of hypoxia‐reoxygenation injury (Sheridan et al. 1991; Mohazzab‐H et al. 1996; Li and Jackson 2002; Ng et al. 2002). However, despite the intensity of the hypoxic insult that was used in our experiments, endothelium‐dependent as well as endothelium‐independent relaxation remained unaltered (Table 1). In contrast to acute hypoxia, chronic hypoxia impaired endothelium‐dependent relaxation in chicken embryo DA (Van der Sterren et al. 2009), FA (Villamor et al. 2004), and MA (Moonen et al. 2012) but not in PA (Villamor et al. 2004). On the other hand, endothelium‐independent relaxation was impaired by chronic hypoxia in chicken DA (Van der Sterren et al. 2009), did not change in femoral and PAs (Villamor et al. 2004) and was increased in MAs (Moonen et al. 2012). Taken together, past and the present data suggest that vascular effects of hypoxia are strongly dependent on the intensity and duration of the insult, the vascular bed, and whether they occur ex or in vivo. In addition, the fetal vessels appear to be particularly resistant to the effects of acute hypoxia‐reoxygenation.

### Significance and perspectives

Species‐ and organ‐specific critical time windows exist during development and, if environmental changes are experienced in the window of vulnerability, the development trajectory of the responding organ may be changed in ways that result in transient or persistent alterations (Nathanielsz 2006). This study shows that a prenatal lethal hypoxic insult did not affect ex vivo vascular oxygen sensing and reactivity in the late chicken embryo. This suggests that acute hypoxia‐induced vascular dysfunction, at least in the model and the four vessels that we have investigated, may not play a relevant role in the pathophysiology of perinatal acute hypoxic cardiovascular injury. Nevertheless, future studies are required to confirm our results in mammalian models of perinatal hypoxia (Strackx et al. 2010). In addition, it remains to be investigated whether exposure of chicken embryos to nonlethal hypoxia‐reoxygenation during different developmental stages would lead to an altered transition to ex ovo life and/or to vascular dysfunction in later stages.

## Conflict of Interest

None declared.
